# Anal Human Papillomavirus Infection among HIV-Infected Men in Korea

**DOI:** 10.1371/journal.pone.0161460

**Published:** 2016-08-22

**Authors:** Chang Hun Lee, Sun Hee Lee, Shinwon Lee, Heerim Cho, Kye-Hyung Kim, Jung Eun Lee, Eun ju Jung, Su jin Lee, Eun Jung Kim, Ki Hyung Kim, Eunsoo Moon, Hong Je Cho

**Affiliations:** 1 Department of Pathology, Pusan National University School of Medicine, Medical Research Institute, Pusan National University Hospital, Busan, Korea; 2 Department of Internal Medicine, Pusan National University School of Medicine, Medical Research Institute, Pusan National University Hospital, Busan, Korea; 3 Department of Internal Medicine, Pusan National University School of Medicine, Yangsan Pusan National University Hospital, Geongsangnam-do, Korea; 4 Department of Obstetrics & Gynecology, Pusan National University School of Medicine, Pusan National University Hospital, Busan, Korea; 5 Department of Psychiatry, Pusan National University School of Medicine, Pusan National University Hospital, Busan, Korea; 6 Department of Surgery, Pusan National University School of Medicine, Pusan National University Hospital, Busan, Korea; Fondazione IRCCS Istituto Nazionale dei Tumori, ITALY

## Abstract

**Background:**

Little is known about the epidemiology on human papillomavirus (HPV) infection among HIV-infected men in Korea. The objective of this study was to determine the prevalence, genotype distribution and risk factors associated with anal HPV infection among HIV-infected men in Korea.

**Methods:**

A single-center cross-sectional study was conducted with HIV-infected men in Korea. Participants completed a detailed sexual behavior risk factor questionnaire. Anal samples were collected for cytology and HPV genotyping. Factors associated with anal HPV infection were assessed using multivariable logistic regression, stratifying by sexual behaviour.

**Results:**

A total of 201 HIV-infected men were included in the study: 133 were from men who have sex with men (MSM) and 68 from men who have sex with women (MSW). Any anal HPV infection was detected in 82.7% of HIV-infected MSM and in 51.5% of HIV- infected MSW (*P* < 0.001). High-risk HPV (HR-HPV) prevalence was higher among MSM (47.4%) than MSW (25.0%; *P* = 0.002). The HR-HPV types identified most frequently were HPV 16 (11%), HPV 18 (9.9%), and HPV 58 (5%) in MSM, and HPV 58(11%) and HPV 16 (8.9%) in MSW. Prevalence of any HPV types in 9-valent vaccine types was higher among MSM than MSW (47.4% vs 22.1%. *P* = 0.001). Abnormal anal cytology was more commonly detected in MSM than MSW (42.9% vs.19.1%, *P* < 0.001). In HIV-infected MSM, higher number of lifetime male sex partners was significantly associated with any anal HPV infection, but age was a significant risk factor associated with anal HR-HPV infection.

**Conclusion:**

Anal HPV infection was highly prevalent in HIV-infected MSM in Korea, and also commonly found in HIV-infected MSW. In HIV-infected MSM, the significant risk factor for being infected with any HPV infection was lifetime number of male sexual partners, and with anal oncogenic HPV infection was age.

## Introduction

Human papillomavirus (HPV), the major cause of cervical cancer, also causes a substantial disease burden in men, including genital warts, penile cancer, anal cancer and certain oropharyngeal cancers. Anal cancer is relatively rare in general population, whereas anal cancer incidence is substantially higher in HIV-infected patients, particularly among men who have sex with men (MSM)[1]. Despite the widespread use of potent antiretroviral therapy (ART), the incidence of anal cancer has not declined, but rather with increased survival, it appears to have increased in HIV-infected patient[2–5].

In Korea, anal cancer is a rare malignancy with an estimated incidence in the general population of 0.5 cases per 100,000 person-years[6, 7], and was also rarely reported among HIV-infected patients[8, 9]. In two separate studies encompassing a total of 1,533 HIV-infected patients of Korea, only one case of anal cancer (0.07%) was reported[8, 9]. Since HIV reporting began in Korea in 1985, a cumulative total of 9,982 Korean people were diagnosed with HIV infection by the end of 2014[7]. The man-to-woman ratio was increased about two times from 7.1:1 in 1997–2001 to 14.3:1 in 2010–2014. Steep increases in the proportion of MSM among the newly diagnosed HIV-infected men were also seen, which doubled in the same period[7].

The prevalence of HPV infection and the burden of HPV-related cancers varied in different geographic areas. With the introduction of HPV vaccine, knowledge regarding high risk-HPV genotype distribution can inform decisions on vaccination strategies for HIV-infected men. To date, there are no data on the prevalence of anal HPV infection in HIV-infected men as well as in general population in Korea

The objectives of this cross sectional study were to assess prevalence and type distribution of anal HPV infection among HIV-infected men in Korea. Risk factors associated with anal HPV infection among MSM were also determined.

## Materials and Methods

### Study population

This cross sectional study was conducted at the Pusan National University Hospital. The hospital is 1,220 bed, university-affiliated teaching hospital and provides HIV care for HIV-infected patients in southeastern region of Korea. We enrolled HIV-infected men who attended the HIV outpatient clinic of the study hospital between July 2014 and January 2015, who were aged ≥18 years, and who had not received a diagnosis of anal cancer prior to enrollment. The protocol was reviewed and approved by the institutional review board (IRB) of Pusan National University Hospital (IRB No. E-2014013). We obtained the written informed consent from all participants before inclusion in the study. The patients who accepted to participate were interviewed using a self-administered questionnaire that included questions on socio-demographic factors, substance use, sexual behavior, and status of circumcision. We also reviewed the medical records to collect the clinical data, and also to compare the reported sexual behavior in questionnaire with those in the medical records. Non-HIV related comorbidity was assessed with Charlson Comorbidity Index (CCI)[10]. We excluded AIDS as a co-morbidity.

### Sample collection, anal cytology and human papillomavirus genotyping

Specimen collection was performed by a health care provider. Anal samples were obtained by introducing a cytobrush blindly into 3 cm into the anal canal and gently rotating it for 30 to 45 seconds in a spiral pattern until the device exits the anal verge[11]. The cytobrush was rinsed in the PreservCyt^®^ solution (Hologic Inc., Boxborough, MA) by rotating the device in the solution 10 times while pushing against the vial wall, and was swirled vigorously to further release material. All samples were sent to the Green Cross Laboratories in Seoul. Cytologic smear results were categorized according to the 2001 Bethesda classification system terminology[12]. HPV genotype was determined using an PCR based DNA microarray system, the HPV DNA chip (HPV Genotypeing Chip^™^ Kit, AGBIO Diagnostics, Seoul, Korea). This contains 32 type specific probes detecting 13 types of high risk HPV (16, 18, 31, 33, 35, 39, 45, 51, 52, 56, 58, 59, and 68) and 19 types of low risk HPV (6, 11, 26, 32, 34, 40, 42, 43, 44, 53, 54, 55, 57, 61, 62, 66, 69, 70 and 73).

### Statistical analysis

The statistical analyses were conducted using PASW Statistics 18 (SPSS Inc., Chicago, IL, USA). Categorical variables were compared using Pearson’s chi-square test or Fisher’s exact test, whereas non-categorical variables were tested with Student t test or the Mann-Whitney U-test. Potential demographic, sexual, behavioral, and other predictors for the HPV infection were analyzed by multivariate logistic regression. All variables with *P* < 0.25 in univariate analysis were assessed in multivariate models using stepwise backward selection. All tests were considered statistically significant at *P* < 0.05.

## Results

### Patients’ characteristics

A total of 133 HIV-infected MSM and 68 HIV-infected MSW were included in this study. Their demographic characteristics are described in Table 1. Median age (interquartile range, IQR) was 46 years old (37–56) for HIV-infected MSM and 50 years old (43–58) for HIV-infected MSW. Among 133 HIV-infected MSM, 81 (60.9%) reported having had sexual intercourses with both men and women. More than 60% of HIV-infected MSM was single compared with 41.2% of HIV-infected MSW (*P* = 0.041). Approximately 34% of HIV-infected MSM had never smoked prior to enrollment compared with 17.6% of HIV-infected MSW (*P* = 0.02). More than half of both HIV-infected MSM and MSW were circumcised (60.9% vs 54.4%). The median baseline CD4 cell count (IQR) among HIV-infected MSM and MSW was 605(486–851) and 594(419–776) cells/mL, respectively. More than 85% of patients of both groups were receiving ART more than 1 year and have undetectable HIV viral load. More than 60% of HIV-infected MSM reported > 5 male sex partners during life, about 50% reported having ≥ 1 male sex partners within 3 months of enrollment. Approximately 47% of HIV-infected MSW reported > 5 lifetime female sex partners, 23.5% reported having ≥ 1 female sex partners during the 3 months before study entry. More than 45% of both groups infrequently used condom.

**Table 1:** Baseline characteristics.

Characteristics	Total population (n = 201)	HIV-infected MSM (n = 133)	HIV-infected MSW (n = 68)	*P* value*
Age, median (IQR), years	48 (39–57)	46 (37–56)	50 (43–58)	0.021
≤ 40	55 (27.4)	43 (32.3)	12 (21.8)	0.059
41–50	51 (25.4)	34 (25.6)	17 (33.3)	
> 50	95 (47.3)	56 (42.1)	39 (57.4)	
Marital status				0.003
Unmarried	113 (56.2)	85 (63.9)	28 (41.2)	
Ever married	88 (43.8)	48 (36.1)	40 (58.8)	
Economy				0.595
High	28 (13.9)	21 (15.8)	7 (10.3)	
Medium	110 (54.7)	71 (53.4)	39 (57.4)	
Low	63 (31.3)	41 (30.8)	22 (32.4)	
Education				0.873
High school or less	140 (69.7)	92 (69.2)	48 (70.6)	
University or more	61 (30.3)	41 (30.8)	20 (29.4)	
Smoking				0.020
Never	57 (28.4)	45 (33.8)	12 (17.6)	
Ever	144 (71.6)	88 (66.2)	56 (82.4)	
History of any psychiatric disorder**				<0.001
No	158 (78.6)	95 (71.4)	63 (92.6)	
Yes	43 (21.4)	38 (28.6)	5 (7.4)	
Charlson comorbidity index				0.219
0	125 (62.2)	87 (65.4)	38 (55.9)	
≥ 1	76 (37.8)	46 (34.6)	30 (44.1)	
CD4 cell counts at the time of HPV test, median (IQR), cells/μL	605 (442–783)	605 (486–851)	594(419–776)	0.222
> 500	129 (64.2)	80 (60.2)	49 (72.1)	0.129
351–500	43 (21.4)	34 (25.6)	9 (13.2)	
≤ 350	29 (14.4)	19 (14.3)	10 (14.7)	
Viral suppression at the time of HPV test				0.217
< 50 copies/mL	181 (90.0)	117 (88.0)	64 (94.1)	
> 50 copies/mL	20 (10.0)	16 (12.0)	4 (5.9)	
ART at the time of HPV test				0.826
> 1 year	176 (87.6)	115 (86.5)	60 (88.2)	
≤ 1 year	25 (12.4)	18 (13.5)	8 (11.8)	
Sexual preference				< 0.001
Heterosexual	68 (33.8)	0 (0)	68 (100)	
Bisexual	81 (40.3)	81 (60.9)	0 (0)	
Homosexual	52 (25.9)	52 (39.1)	0 (0)	
Circumcision				0.449
No	83 (41.3)	52 (39.1)	31 (45.6)	
Yes	118 (58.7)	81 (60.9)	37 (54.4)	
Numbers of lifetime female sex partners				< 0.001
0	52 (25.9)	52 (39.1)	0 (0)	
1–5	102 (50.7)	66 (49.6)	36 (52.9)	
> 5	47 (23.4)	15 (11.3)	32 (47.1)	
Numbers of lifetime male sex partners				< 0.001
1–5	48 (23.9)	48 (36.1)	0 (0)	
6–10	22 (10.9)	22 (16.5)	0 (0)	
> 10	63 (31.3)	63 (47.4))	0 (0)	
Any female sex partners in last 3 months				0.006
No	173 (86.1)	121 (91.0)	52 (76.5)	
Yes	28 (13.9)	12 (9.0)	16 (23.5)	
Any male sex partners in last 3 months				< 0.001
No	136 (67.7)	68 (51.1)	68 (100)	
Yes	65 (32.3)	65 (48.9)	0 (0)	
Unprotected sexual intercourse				0.882
Sometimes	108 (53.7)	72 (54.1)	36 (52.9)	
Frequently	93 (46.3)	61 (45.9)	32 (47.1)	
Self-reported history of STI	119 (59.2)	84 (63.2)	35 (51.5)	0.130
Gonorrhea	48 (23.9)	32 (24.1)	16 (23.5)	1.000
Nonspecific urethritis	23 (11.4)	19 (14.3)	4 (5.9)	0.101
Syphilis	65 (32.3)	50 (37.6)	15 (22.1)	0.038
Genital herpes	5 (2.5)	4 (3.0)	1 (1.5)	0.664
Genital warts	9 (4.5)	7 (5.3)	2 (2.9)	0.721
Pediculosis pubis	65 (32.3)	50 (37.6)	15 (22.1)	0.038
Syphilis seropositive	105 (52.2)	74 (56.5)	31 (44.3)	0.106
HBV seropositive	20 (10.0)	13 (9.8)	7 (10.3)	1.000
HCV seropositive	6 (3.0)	3 (2.3)	3 (4.4)	0.666

Data are number (%) of patients, unless otherwise indicated. IQR, interquartile range; HIV, human immunodeficiency virus; MSM, men who have sex with men; MSW, men who have sex with women; HPV, human papillomavirus; ART, antiretroviral therapy; STI, sexual transmitted infection; HBV, hepatitis B virus; HCV, hepatitis C virus;

*Calculated using χ^2^ test, Fisher’s exact test, and t-test.;

** History of any of any illicit durg use was included

### Anal HPV prevalence

Overall, HPV infection rate was significantly higher in HIV-infected MSM than HIV-infected MSW (82.7% vs 51.5%, *P* < 0.001). Multiple HPV types were more frequently detected among HIV-infected MSM compared with HIV-infected MSW (24.8% vs 11.8%, *P* = 0.041). Prevalence of HR-HPV infection was significantly higher among HIV-infected MSM than among HIV-infected MSW (47.4% vs 25.0%, *P* = 0.002). Multiple HR-HPV types were also frequently detected in HIV-infected MSM compared with HIV-infected MSW (13.5% vs 1.5%, *P* = 0.001).

**Fig 1:**
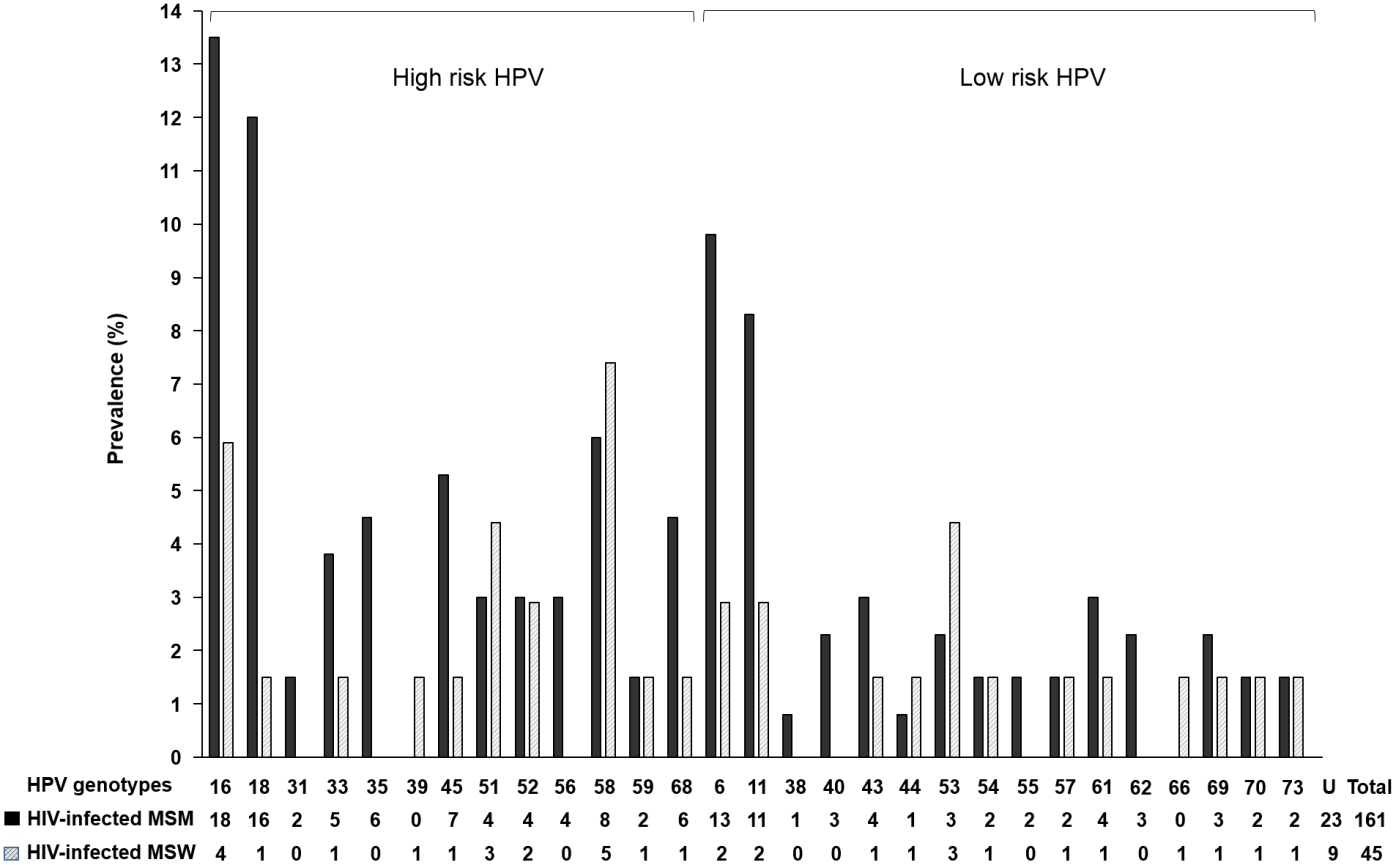
Human papillomavirus (HPV) genotypic distribution among HIV-infected Men who have sex with men and among HIV-infected men who have sex with women. (MSM, men who have sex with men; MSW, men who have sex with women; U, untypeable)

Fig 1 shows the type specific HPV prevalence of both HIV-infected MSM and HIV-infected MSW. In HIV-infected MSM, the most commonly detected HR-HPV genotypes were HPV 16 (11%), followed by 18 (9.9%), 58 (5%), 45 (4.3%), HPV 35 (3.7%), 68 (3.7%) and 33 (3.1%). In HIV-infected MSW, the most prevalent HR-HPV was HPV 58 (11%), followed by 16 (8.9%), 51 (6.7%) and 52 (4.4%). Commonly encountered non-oncogenic virus was HPV 6 (8.1%), 11 (6.8%), 43 (2.5%) and 61 (2.5%) in HIV-infected MSM, and HPV 53 (6.7%), 6 (4.4%) and 11 (4.4%) in HIV-infected MSW, respectively.

The prevalence of vaccine preventable HPV types in bivalent (HPV 16/18), quadrivalent (HPV 6/11/16/18) and 9-valent (HPV 6/11/16/18/31/33/45/52/58) in anal sample was higher in HIV-infected MSM (22.6%, 36.8%, 47.4%) than MSW (7.4%, 10.3%, 22.1%) (Table 2).

**Table 2:** Prevalence of Human papillomavirus (HPV) infection and abnormal anal cytology.

Variables	Men who have sex with men (n = 133)	Men who have sex with women (n = 68)	*P* value*
Any HPV positive	110 (82.7)	35 (51.5)	< 0.001
Multiple types of any HPV	33 (24.8)	8 (11.8)	0.041
HR-HPV positive	63 (47.4)	17 (25.0)	0.002
Multiple types of HR-HPV	18 (13.5)	1 (1.5)	0.004
Any 2vHPV types positive	30 (22.6)	5 (7.4)	0.006
Multiple 2vHPV types	4 (3.0)	0 (0)	0.302
Any 4vHPV types positive	49 (36.8)	7 (10.3)	< 0.001
Multiple 4vHPV types	10 (7.5)	1 (1.5)	0.103
Any 9vHPV types positive	63 (47.4)	15 (22.1)	0.001
Multiple 9vHPV types	19 (14.3)	2 (2.9)	0.014
Abnormal anal cytology	57 (42.9)	13 (19.1)	0.001
ASCUS-US	26 (19.5)	4 (5.9)	
LSIL	30 (22.6)	9 (13.2)	
HSIL	1 (0.8)	0 (0)	

Data are number (%) of patients, unless otherwise indicated. HIV, human immunodeficiency virus; HPV, human papilloma virus; HR, high risk; 2vHPV, bivalent human papillomavirus vaccine; 4vHPV, quadirivalent human papilloma virus vaccine; 9vHPV, 9-valent human papilloma virus vaccine; ASCUS-US, atypical squamous cells of undetermined significance; LSIL, low-grade squamous intraepithelial lesions; HSIL, high-grade squamous intraepithelial lesions;

* Calculated using χ^2^ test, Fisher’s exact test.

### Anal cytology

A total of 57 HIV-infected MSM (42.9%) and 13 HIV-infected MSW (19.1%) had abnormal cytology (Table 2). Eighty HIV-infected individuals with HR-HPV were more likely to have abnormal cytology than those without HR-HPV (47.5% vs 26.4%, *P* = 0.002). When comparing 63 HIV-infected MSM with HR-HPV with 17 HIV-infected MSW with HR-HPV, there was no difference of having abnormal cytology between both groups (47.6% vs 47%). Of the 36 HR-HPV detected from 40 HIV-infected individuals with HSIL or LSIL, the most frequently detected HR-HPV was HPV 16(22.2%) and 18(22.2%), followed by 51(16.7%), 33(11.1%), 45(8.3%), 58(8.3%), 52(5.6%), 56(2.8%) and 68(2.8%).

### Risk factors associated with anal HPV infection among HIV-infected MSM

In multivariate analyses, higher number of lifetime male sex partners was significantly associated with anal any HPV infection among HIV-infected MSM [in comparison to 1–5 partners: adjusted odds ratio (aOR) 1.85, 95% confidence interval(CI) 0.53–6.46 for 1–6 partners, aOR 4.78, 95% CI 1.58–14.43 for ≥ 10 partners, *P* = 0.021] (Table 3).

**Table 3:** Univariable and multivariable analyses of determinants of anal human papillomavirus infection in HIV-infected Men who have sex with men.

Characteristics	HPV infection among HIV-infected MSM (n = 133)				
	HPV	Univariate		Multivariate	
	No.	OR(95% CI)	*P* value*	OR(95% CI)	*P* value*
Age, years			0.396		-
≤ 40	38 (88.4)	1		-	
41–50	26 (76.5)	0.43 (0.13–1.45)		-	
> 50	46 (82.1)	0.61 (0.19–1.92)		-	
Economy			0.869		-
High	18 (85.7)	1		-	
Medium	59 (83.1)	0.82 (0.21–3.23)		-	
Low	33 (80.5)	0.69 (0.16–2.92)		-	
Marital status			0.739		-
Unmarried	71 (83.5)	1		-	
Ever married	39 (81.3)	0.85 (0.34–2.15)		-	
Education			0.304		-
High school or less	74 (80.4)	1		-	
University or more	36 (87.8)	1.75 (0.60–5.10)		-	
Smoking			0.705		-
Never	38 (84.4)	1		-	
Ever	72 (81.8)	0.83 (0.31–2.19)		-	
History of any psychiatric disorder**			0.428		-
No	77 (81.1)	1		-	
Yes	33 (86.8)	1.54 (0.53–4.51)		-	
Charlson comorbidity index			0.983		-
0	72 (82.8)	1		-	
≥ 1	38 (82.6)	0.99 (0.39–2.54)		-	
CD4 cell counts at the time of HPV test, median (IQR), cells/μL			0.858		-
> 500	65 (81.3)	1		-	
351–500	29 (85.3)	1.34 (0.44–4.03)		-	
≤ 350	16 (84.2)	1.23 (0.32–4.77)		-	
Viral suppression at the time of HPV test			0.870		-
< 50 copies/mL	97 (82.9)	1		-	
> 50 copies/mL	13 (81.3)	0.89 (0.23–3.43).		-	
ART at the time of HPV test			0.188		-
> 1 year	93 (80.9)	1		-	
≤ 1 year	17 (94.4)	4.02(0.51–31.86)		-	
Sexual preference			0.636		-
Bisexual	68 (84.0)	1		-	
Homosexual	42 (80.8)	0.80 (0.32–1.99)		-	
Being circumcised			0.162		-
No	40 (76.9)	1		-	
Yes	70 (86.4)	1.91 (0.77–4.72)		-	
Numbers of lifetime female sex partners			0.448		-
0	41 (78.8)	1		-	
1–5	55 (83.3)	1.34 (0.53–3.40)		-	
>5	14 (93.3)	3.76 (0.44–31.77)		-	
Any female sex partners in last 3 months			0.952		-
No	100 (82.6)	1		-	
Yes	10 (83.3)	1.05 (0.21–5.15)		-	
Numbers of lifetime male sex partners			0.021		0.021
1–5	34 (70.8)	1		1	
6–10	18 (81.8)	1.85 (0.53–6.46)		1.85 (0.53–6.46)	
>10	58 (92.1)	4.78 (1.58–14.43)		4.78 (1.58–14.43)	
Any male sex partners in last 3 months			0.570		-
No	55 (80.9)	1		-	
Yes	55 (84.6)	1.30 (0.53–3.21)		-	
Unprotected sexual intercourse			0.244		-
Sometimes	57 (79.2)	1		-	
Frequently	53 (86.9)	1.74 (0.68–4.45)		-	
Self-reported history of STI			0.803		-
No	40 (81.6)	1		-	
Yes	70 (83.3)	1.13 (0.45–2.83)		-	
Syphilis serology			0.150		-
Negative	44 (77.2)	1		-	
Positive	66 (86.8)	1.95 (0.79–4.84)		-	
HBV/HCV serology			0.591		-
Negative	96 (82.1)	1		-	
Positive	14 (87.5)	1.53 (0.32–7.25)		-	

Data are number (%) of patients, unless otherwise indicated. HIV, human immunodeficiency virus; HPV, human papilloma virus; ART, antiretroviral therapy; STI, sexual transmitted infection; HBV, hepatitis B virus; HCV, hepatitis C virus;

*Calculated using χ^2^ test, Fisher’s exact test, and multi-logistic regression analysis.

** History of any of any illicit drug use was included

In multivariate analyses for detection of any anal HR-HPV infection in HIV-infected MSM, age was only significant risk factor (in comparison to ≤ 40 years old: aOR 0.27, 95% CI 0.10–0.76 for 41–50 years old, aOR 0.14, 95% CI 0.05–0.41 for > 50 years old, *P* = 0.002) (Table 4).

**Table 4:** Univariable and multivariable analyses of determinants of high risk human papillomavirus infection in HIV-infected Men who have sex with men.

Characteristics	HR HPV infection in HIV-infected MSM (n = 133)				
	HPV	Univariate		Multivariate	
	No.	OR(95% CI)	*P* value*	OR(95% CI)	*P* value*
Age, years			0.021		0.001
≤ 40	28 (65.1)	1		1	
41–50	13 (38.2)	0.33 (0.13–0.84)		0.27 (0.10–0.76)	
> 50	22 (39.3)	0.35 (015–0.79)		0.14 (0.05–0.41)	
Economy			0.313		-
High	13 (61.9)	1		-	
Medium	33 (46.5)	0.53 (0.20–1.45)		-	
Low	17 (41.5)	0.44 (0.15–1.28)		-	
Marital status			0.790		-
Unmarried	41 (48.2)	1		-	
Ever married	22 (45.8)	0.91 (0.45–1.85)		-	
Education			0.333		-
High school or less	41 (48.2)	1		-	
University or more	22 (45.8)	1.44 (0.69–3.02)		-	
Smoking			0.908		-
Never	21 (46.7)	1		-	
Ever	42 (47.7)	1.04 (0.51–2.14)		-	
History of any psychiatric disorder**			0.701		-
No	44 (46.3)	1		-	
Yes	19 (50.0)	1.16 (0.55–2.46)		-	
Charlson comorbidity index			0.514		-
0	43 (49.4)	1		-	
≥ 1	20 (43.5)	0.79 (0.38–1.62)		-	
CD4 cell counts at the time of HPV test, median (IQR), cells/μL			0.591		-
> 500	35 (43.8)	1		-	
351–500	18 (52.9)	1.45 (0.65–3.24)		-	
≤ 350	10 (52.6)	1.43 (0.52–3.90)		-	
Viral suppression at the time of HPV test			0.822		-
< 50 copies/mL	55 (47.0)	1		-	
> 50 copies/mL	8 (50.0)	1.13 (0.40–3.21)		-	
ART at the time of HPV test			0.214		-
> 1 year	52 (45.2)	1		-	
≤ 1 year	11 (61.1)	1.90 (0.69–5.26)		-	
Sexual preference			0.197		-
Bisexual	42 (51.9)	1		-	
Homosexual	21 (40.4)	0.63 (0.31–1.27)		-	
Being circumcised			0.896		-
No	25 (48.1)	1		-	
Yes	38 (46.9)	0.95 (0.48–1.92)		-	
Numbers of lifetime female sex partners			0.260		-
0	20 (38.5)	1		-	
1–5	35 (53.0)	1.81 (0.86–3.78)		-	
>5	8 (53.3)	1.83 (0.57–5.82)		-	
Any female sex partners in last 3 months			0.171		-
No	55 (45.5)	1		-	
Yes	8 (66.7)	2.40 (0.69–8.40)		-	
Numbers of lifetime male sex partners			0.820		-
1–5	21 (43.8)	1		-	
6–10	11 (50.0)	1.29 (0.47–3.54)		-	
>10	31 (49.2)	1.25 (0.59–2.65)		-	
Any male sex partners in last 3 months			0.534		-
No	34 (50.0)	1		-	
Yes	29 (44.6)	0.81 (0.41–1.59)		-	
Unprotected sexual intercourse			0.154		-
Sometimes	30 (41.7)	1		-	
Frequently	33 (54.1)	1.65 (0.83–3.28)		-	
Self-reported history of STI			0.776		-
No	24 (49.0)	1		-	
Yes	39 (46.4)	0.90 (0.45–1.83)		-	
Syphilis serology			0.726		-
Negative	28 (49.1)	1		-	
Positive	35 (46.1)	0.88 (0.44–1.76)		-	
HBV/HCV serology					-
Negative	58 (49.6)	1	0.176	-	
Postive	5 (31.3)	0.46 (0.15–1.41)		-	

Data are number (%) of patients, unless otherwise indicated. HIV, human immunodeficiency virus; HPV, human papilloma virus; ART, antiretroviral therapy; STI, sexual transmitted infection; HBV, hepatitis B virus; HCV, hepatitis C virus;

*Calculated using χ^2^ test, Fisher’s exact test, and multi-logistic regression analysis.

** History of any of any illicit durg use was included

## Discussion

To our knowledge, this is the first report of anal HPV prevalence among HIV-infected men in Korea. In this study, the prevalence of anal HPV infection with the 32 virus types tested and HR-HPV infection among HIV-infected MSM was 82.7% and 47.4%, respectively. Although there can be some differences in the prevalence of anal HPV infection across different studies according to the sampling methods and diagnostic methods[13], this is consistent with the results from the earlier studies conducted in HIV-infected MSM[14–28].

There are few published studies comparing anal HPV infection among HIV-infected MSM and MSW[29–35]. Previous studies have shown that anal HPV infection are more prevalent among HIV-infected MSM than among HIV-infected MSW (84–85.6% vs 40.8–46%)[29, 30, 33]. HIV-infected MSM also had a higher prevalence of HR-HPV compared with HIV-infected MSW (54–75% vs 18–28%)[31, 32, 34]. In our study, the prevalence of both anal HPV infection (82.7% vs 51.5%) and oncogenic anal HPV infection (47.4% vs 25%) were also significantly higher in HIV-infected MSM than HIV-infected MSW.

Similar to previous studies[19–21, 25, 28], the most commonly detected HR-HPV types in HIV-infected MSM in our study were HPV 16 and 18, which are the types most commonly associated with anal cancer. In contrast to HIV-infected MSM, the most prevalent HR-HPV in HIV-infected MSW was HPV 58, which has been found to be prevalent among patients with cervical cancer in East Asia[36]. The type-specific prevalence of anal HPV infection has ranged widely in studies among HIV-infected men in East Asia[19, 20, 23, 28, 31, 37]. In HIV-infected MSM, HPV-16 was the most frequently detected HR-HPV types both in China (14.3–34%)[19, 20, 23, 37] and in Taiwan (10%)[28], while HPV-58 was most commonly found in Japan (30.2%)[31]. In HIV-infected MSW, although there are limited data in East Asia due to small number of subjects, HPV-16 was the most prevalent HR-HPV types both in Taiwan (15.6%)[28] and in Japan (14.7%)[31]. On the other hand, HPV-58, the most common HR-HPV type of HIV-infected MSW in our study, was infrequently detected both in Taiwan (1.6%)[28] and in Japan (2.9%)[31]. Consistent with our results, HPV-6 and HPV-11 were consistently among the most common LR-HPV types detected in both MSM and MSW across studies regardless of the geographic locales[19, 20, 23, 28, 31, 37]. In the present study, significantly more HIV-infected MSM (24.8%) than HIV-infected MSW (11.8%) had multiple HPV infections, findings were broadly in line with those in previous studies. In Taiwan, multiple HPV infection was observed in 6.2% of HIV-infected MSM and in 4.7% of HIV-infected MSW[28]. In Japan, compared to HIV-infected MSW (14.7), multiple HR-HPV infection was more commonly found in HIV-infected MSM (30.2)[31].

In this study, overall prevalence of vaccine-preventable HPV types in anal specimens of HIV-infected men was 17.4%, 27.9%, and 38.8% for the 2vHPV, 4vHPV and 9vHPV types, respectively. The prevalence was much higher in HIV-infected MSM (respectively 22.6%, 36.8% and 47.4%) than in HIV-infected MSW (respectively 7.4%, 10.3%, and 22.1%). None had all 4vHPV or 9vHPV types detected. These vaccine-preventable type prevalence may be underestimated because a cross-sectional design with HPV DNA testing detect current infection but gives no information on previous resolved HPV infections[38]. This indicate significant proportion of HIV-infected MSM as well as HIV-infected MSW have already acquired HPV vaccine types, which may reduce the benefit of vaccination.

In HIV-infected MSM, several risk factors, including number of sex partners[21, 28, 39], receptive anal intercourse[21, 39], and unprotected oral sex[20] have been suggested as a predictors of anal HPV infection. Consistent with previous studies, lifetime numbers of male sex partner was a main predictor for anal HPV infection in HIV-infected MSM in our study[21, 28, 39]. The patients having more than 10 male sexual partners in their lifetime were about 4.8 times more likely to have HPV infection in anus than those having less than 5 partners. In the previous studies investigating risk factors for anal HR-HPV infection in HIV-infected MSM, reported risk factors for anal HR-HPV infection varied across different studies. They included lower education[20], smoking[17, 40], younger age[15, 18, 31], lower CD4 cell counts[18, 31, 40, 41], higher nadir CD4 cell counts[15], number of sex partners, receptive anal intercourse[15, 28], having sexual transmitted infections (STIs)[31, 40]. In our study, age was a significant risk factor for anal HR-HPV in HIV-infected MSM. These findings are consistent with other studies that have shown that younger age was a significant risk factors for anal HR-HPV[15, 18, 31]. This finding may results from age-specific sexual behavior. In our study, the proportion of having any male sex partners in last 3 months was higher in MSM ≤ 40 years of age (53.5%) compared with HIV-infected MSM aged > 50 years (37.5%).

In the present study, we also found high prevalence of abnormal anal cytology in both HIV-infected MSM (42.9%) and HIV-infected MSW (19.1%). Our results are similar to those of previous studies in that HIV-infected MSW also have substantial prevalence of abnormal anal cytology[29–34], suggesting that anal cancer screening needs to be considered in HIV-infected MSW.

This study has some limitations. First, this study was a cross-sectional study. The longitudinal follow-up data were not available. Second, our study was conducted in a single center, therefore our findings may not be generalized to other region of the country. Third, we did not test all genotypes of HPV, and the proportion of persons with untypeable HPV was relatively high. Forth, our study was designed to assess the cytology, so we did not include the histologic findings. Fifth, data on socio-demographic and risk behaviors collected by self-administered questionnaire may have potential information bias caused by inaccurate response. We also did not include some risky sexual behaviors as variables, such as anal sex history. Sixth, we were not able to assess the risk factors for anal HPV infection in HIV-infected MSW, because of insufficient number of enrolled subjects. Even with these limitations, our study could reflect the overall situation of HPV infection among HIV-infected men in Korea.

In conclusion, although anal cancer was rarely reported in HIV-infected men in Korea, high prevalence of HPV infection was observed in HIV-infected MSM, and also commonly found in HIV-infected MSW. Lifetime number of male sex partner and younger age were main predictors for anal any HPV infection and anal HR-HPV infection in HIV-infected MSM, respectively. Although public health programs for prevention of cervical cancer, such as national cervical cancer screening program (since 1999) and national HPV vaccination program for 12-year-old girls (since June, 20016), has been conducted, there is no effective program currently available for anal cancer prevention in Korea. Public health program to promote knowledge about HPV infection and anal cancer, particularly in risk groups including MSM, are needed in Korea. Further studies are also needed to determine the predictors for anal HPV infection in HIV-infected MSW.

## Supporting Information

S1 Dataset (XLSX)
